# Muscle wasting: A review of exercise, classical and non‐classical RAS axes

**DOI:** 10.1111/jcmm.14412

**Published:** 2019-07-05

**Authors:** Mark A. Winslow, Stephanie E. Hall

**Affiliations:** ^1^ Department of Kinesiology Boise State University Boise Idaho

**Keywords:** Angiotensin 1‐7, Angiotensin II, exercise, fibrosis, protein synthesis, ROS, UPS

## Abstract

This review identifies how the classical/non‐classical renin‐angiotensin system (RAS) and exercise influence muscle wasting. The classical RAS axis enhances muscle loss through the interaction with NADPH oxidase (NOX), ubiquitin proteasome system (UPS), protein synthesis and fibrosis pathways. The mainstream hypothesis identifies reactive oxygen species (ROS) as the key pathway in muscle, this review recognizes alternative pathways that lead to an increase in muscle wasting through the classical RAS axis. In addition, pathways in which the non‐classical RAS axis and exercise inhibit the classical RAS axis are also explored. The non‐classical RAS axis and exercise have a significant negative impact on ROS production and protein synthesis. The non‐classical RAS axis has been identified in this review to directly affect protein synthesis pathways not by altering the pre‐existing intracellular ROS level, further supporting the idea that muscle wasting caused by the classical RAS system is not entirely due to ROS production. Exercise has been identified to modify the RAS axes making it a therapeutic option.

## 1 INTRODUCTION

The renin‐angiotensin system (RAS) is important in regulating of blood pressure. The latest research shows these axes are key players in skeletal muscle balance.^1^, ^2^, ^3^, ^4^ The classical RAS axis involves the conversion of angiotensin I (Ang‐I) into angiotensin II (Ang‐II) by angiotensin‐converting enzyme (ACE).^1^ Ang‐II has two primary receptors: Ang‐II type 1 receptor (AT_1_R) and Ang‐II type 2 receptor (AT_2_R).^2^ Ang‐II/AT_1_R signal transduction causes a pathological cascade resulting in increased reactive oxygen species (ROS), protein degradation, fibrosis and a decrease in protein synthesis within skeletal muscle.^3^, ^4^, ^5^, ^6^ Ang‐II/AT_2_R signal transduction has been identified with the differentiation and regenerative processes of myoblasts, which indicates that the activation of AT_2_R may oppose the actions of AT_1_R activation.^7^, ^8^

The non‐classical RAS axis displays protective effects by inhibiting Ang‐II/AT_1_R‐induced pathogenesis.^9^ This non‐classical axis involves the conversion of Ang‐II into angiotensin 1‐7 (Ang 1‐7) by angiotensin‐converting enzyme 2 (ACE 2).^10^ Recent research suggests Ang 1‐7 signalling, mediated by the Mas receptor (MasR), results in a therapeutic cascade, antagonizing Ang‐II‐induced pathogenesis.^11^ Exercise has been implicated in the inhibition of Ang‐II‐induced pathogenesis by directly upregulating the non‐classical RAS axis or by directly inhibiting Ang‐II‐induced pathological mechanisms within skeletal muscle.^10^, ^12^ Mechanisms of Ang 1‐7 and exercise within skeletal muscle are not as well‐known as Ang‐II mechanisms but research implicates these pathways as a possible therapeutic approach to Ang‐II‐induced pathologies.

This review investigates pathological and therapeutic mechanisms induced by the RAS axes and exercise within skeletal muscle. To accomplish this, the review of literature is organized below by each pathway of interest: Oxidative stress, Ubiquitin Proteasome System (UPS), Protein Synthesis and Fibrosis. The review will also provide insight into possible protein targets for future research and therapeutic products.

## 2 SECTION 1: REACTIVE OXYGEN SPECIES

### 2.1 Angiotensin II‐induced ROS production

Oxidative stress is one of the most studied mechanisms of skeletal muscle atrophy. It is the result of increased reactive oxygen species (ROS) production in skeletal muscle leading to atrophy through multiple mechanisms such as inducing apoptosis, increasing proteolysis and decreasing protein synthesis.^5^, ^13^, ^14^, ^15^, ^16^, ^17^ Ang‐II‐mediated increase in muscle ROS production occurs, at least in part, due to AT_1_R‐mediated increases in both NADPH oxidase (NOX) activity and elevated mitochondrial ROS production leading to the speculation that NOX/mitochondrial cross‐talk exists in skeletal muscle exposed to high levels of Ang‐II.^1^, ^13^, ^18^ Regardless of the cellular site(s) of Ang‐II‐induced ROS production in skeletal muscles, it is established that oxidative stress contributes to inactivity‐induced muscle atrophy by increasing proteolysis, increasing myonuclear apoptosis and depressing protein synthesis.^19^ After 4 weeks of Ang‐II infusion, mice lacking the expression of NOX2 exhibited no skeletal muscle atrophy.^5^ However, the wild‐type mice infused with Ang‐II exhibited skeletal muscle atrophy.^5^ This study also indicates that skeletal muscle apoptosis may not be caused by NOX2‐derived ROS because the only group not showing an increase in apoptosis was the wild type infused vehicle mice.^5^ This study suggests that there are alternative ROS that may only activate certain pathways.

ROS can act upon multitude pathways, one of which leads to impaired glucose uptake and desensitization of insulin within skeletal muscle.^20^ Impaired glucose uptake and insulin sensitivity cause a decline in adenosine triphosphate (ATP) production, which creates an imbalance between protein degradation and synthesis.^13^, ^21^ When ATP is in short supply, protein degradation increases to scavenge necessary products to make ATP.^22^ This mechanism has two possible pathways and both involve the interaction with protein kinase B (Akt). Akt, when activated, initiates protein synthesis and can inhibit protein breakdown.^23^ ROS inhibits phosphorylation of Akt and decreases this protein's activity causing a decrease in skeletal muscle protein synthesis and increase in protein degradation.^4^, ^5^ By decreasing Akt activity, glucose transporter 4 (GLUT4) translocation is inhibited resulting in decreased glucose uptake and insulin sensitivity.^20^ The other pathway leading to the inhibition of GLUT4 translocation is caused by the increase in nuclear factor kappa‐light‐chain‐enhancer of activated B cells (NF‐κB) initiated by ROS.^18^ NF‐intracellular directly acts upon Akt and decreases its activity; Akt inhibition leads to an increase in NF‐intracellular, which further inhibits Akt and continuously drives the inhibition of GLUT4 translocation.^18^ This mechanism involving NF‐intracellular provides the first feedback loop in the Ang‐II‐induced pathological state.

Mitochondrial dysfunction can also be attributed to Ang‐II and increased intracellular ROS.^1^, ^15^ ROS has been cited for causing an increase in p38 mitogen‐activated protein kinase (p38MAPK) activity, triggering mitochondrial dysfunction linked to altered electron transport chain (ETC) complex I/III and state 3 respiration.^4^, ^5^, ^17^ Declines in ETC complex I/III and state 3 respiration result in an ATP deficiency, which can be associated with a reduction in both muscle fibre type I and type II occurs causing a reduction in cross‐sectional area (CSA). Ang‐II also reduces peroxisome proliferator‐activated receptor gamma coactivator 1‐alpha (PGC‐1α) activity, which enhances the oxidative capacity of muscle fibres leading to a decline in exercise capacity.^1^, ^4^, ^15^ Continuous mitochondrial dysfunction can lead to its destruction, characterized in these pathological states by a decrease in skeletal muscle mitochondria and ultimately leads to skeletal muscle atrophy.

**Figure 1:**
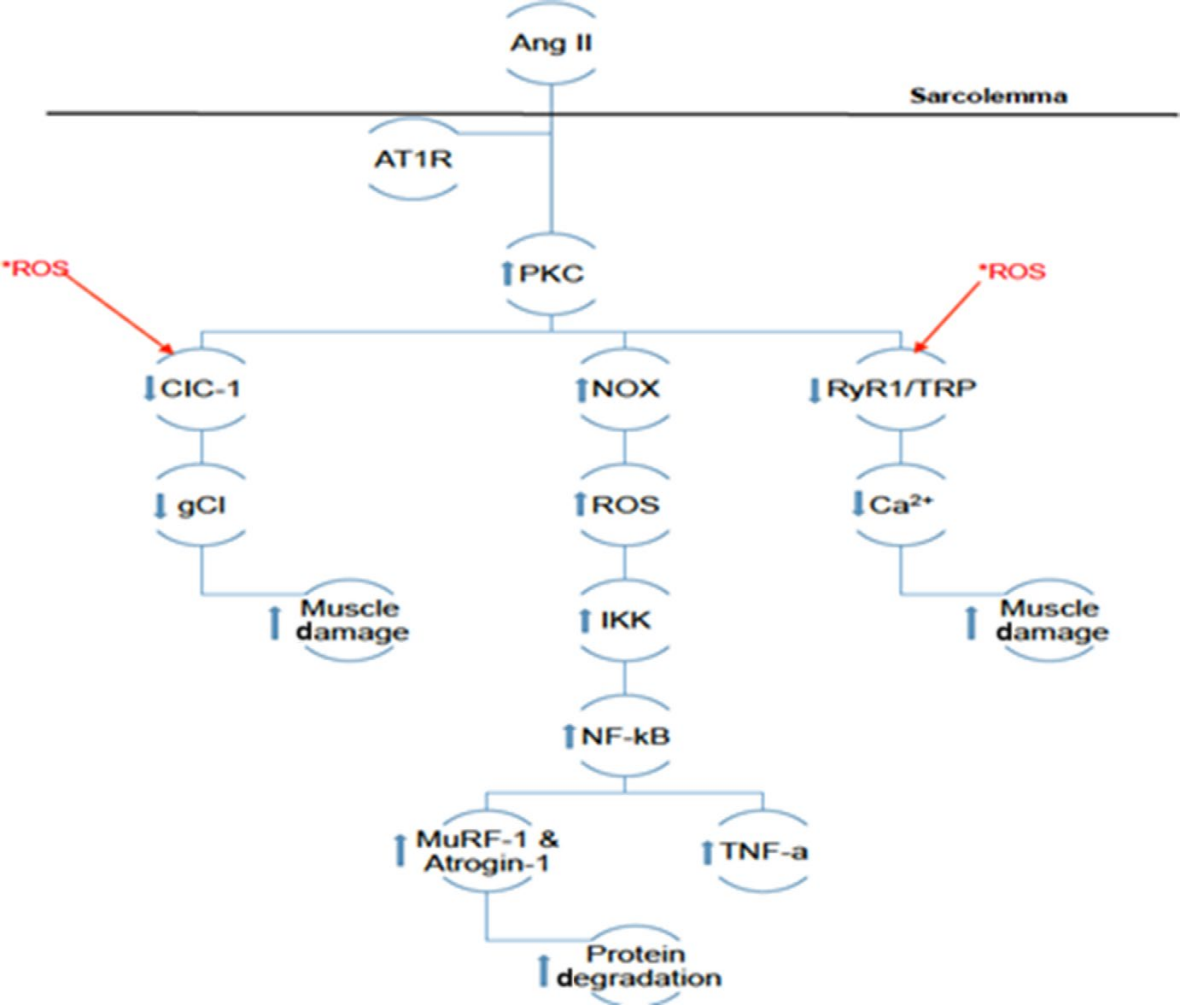
Depicts the theoretical pathway of Ang‐II‐induced increase in ROS production and muscle damage. *ROS indicates the presence of intracellular ROS created by this pathway and natural pre‐existing forms. *ROS and the arrows originating from them indicate proteins intracellular ROS can interact with and illustrate the feedback loops mentioned in section 1. Other arrows illustrate the up‐regulation or down‐regulation of intracellular proteins by Ang‐II mediated by AT1R

ROS can cause apoptosis of skeletal muscle by disrupting membrane chlorine conductance (gCl) and Ca^2+^ channels.^24^ Cozzoli 14 identified Ang‐II/AT_1_R‐induced increase in protein kinase C (PKC) activity upstream of NOX and the increase in ROS leading to a decrease of gCl and increase in intracellular Ca^2+^. The increase in Ca^2+ ^was due to ROS interacting with ryanodine receptor 1 (RyR1) and transient receptor potential (TRP), allowing Ca^2+^ to flow uninhibited from sarcoplasmic reticulum and extracellular fluid.^14^ The decrease in Cl^−^is caused by ROS and PKC inhibiting the CIC‐1 channel, preventing Cl^‐^ to enter the cell.^14^, ^25^ This process decreases cell membrane potential, allowing muscle to constantly contract, eventually leading to damage and apoptosis. Muscle damage and loss associated with Ang‐II can be characterized by the decrease in myosin heavy chain (MHC) which is commonly seen in Ang‐II pathogenesis.^2^ Figure 1 illustrates previously mentioned mechanisms of Ang‐II‐induced ROS production and two muscle damaging pathways that ROS affects.

Degradation of mitochondria and skeletal muscle can initiate proteolysis machinery within the ubiquitin proteasome system. This system can be initiated by ROS and Ang‐II, the mechanisms involved in the upregulation of proteolysis will be described in Section 2.

### 2.2 Angiotensin 1‐7 may decrease ROS production and resulting oxidative damage

Ang 1‐7 is known to antagonize Ang‐II, yet the mechanisms involved in Ang 1‐7's inhibition of Ang‐II remain unclear. The influence of Ang 1‐7 on intracellular activity is mediated by MasR^26^, ^27^, ^28^ and some reports suggest that Ang 1‐7 might act on the primary Ang‐II receptor AT_1_R.^29^ Ang 1‐7 actions to prevent ROS‐related damage are associated with the increase in insulin sensitivity/glucose uptake, preventing MHC decline, decline in apoptosis and inhibition of ROS.^2^, ^9^, ^28^, ^29^, ^30^

As described in the previous section, Ang‐II decreases Akt activity leading to the inhibition of GLUT4 translocation.^18^ Ang‐II can directly cause the inhibition of GLUT4 relocation by inhibiting activated protein kinase (AMPK) activity by increasing protein phosphatase 2C alpha (PP2Cα) 31 or through the production of NF‐κB and ROS, which directly inhibits Akt activity, which is needed for GLUT4 relocation.^5^, ^32^ Ang 1‐7's ability to increase Akt activity could be the result of declines in ROS. Research indicates that Ang 1‐7 does not decrease pre‐existing ROS levels, suggesting that Ang 1‐7 directly inhibits NOX activity.^9^, ^29^, ^32^ Although it seems that ROS is responsible for the initiation of previously described pathogenic pathways, one cannot rule out the other proteins that decrease Akt activity, such as NF‐κB and caspase‐3. With that in mind, Ang 1‐7‐induced increase in glucose uptake and insulin sensitivity could be a result of the inhibition of NF‐κB, caspase‐3 and ROS production.^30^, ^33^ The ability to distinguish mechanisms not associated with ROS is difficult. NOX has been suggested as the primary ROS producer by experiments inhibiting NOX, resulting in the decline of the aforementioned mechanisms 3, 14, 18; however, mitochondrial dysfunction also leads to ROS production, which can reestablish these pathological pathways, establishing the second feedback loop.^15^, ^16^, ^34^ Does Ang 1‐7/MasR axis directly inhibit NOX?

Based on Ang 1‐7‐induced ability to stabilize force reduction and loss of MHC,^2^ Ang 1‐7 likely inhibits the interaction between AT_1_R and PKC. As previously mentioned, PKC has been suggested as a stimulator of NOX, resulting in ROS. Both PKC and ROS are associated with increases in muscle damage, decline in force production and MHC, resulting in a decline in muscle CSA and fibre‐type alterations.^14^, ^35^ Ang 1‐7 prevents these alterations from happening, which suggests that Ang 1‐7 could inhibit Ang‐II/AT_1_R‐induced activation of PKC. In fact, one report found that Ang 1‐7/MasR activity leads to a decrease in AT_1_R expression.^2^ This partially explains the actions of Ang 1‐7 but the mechanism remains elusive, as the increase of MasRs may be dependent on AT_1_R.^36^ Morales^36^ using a rat model infused with Ang‐II, lipopolysaccharide (LPS) and unilateral immobilization saw an increase in Mas receptors. This increase was AT_1_R dependent, as treatment with losartan (AT_1_R blocker) prevented increases in Mas receptors.^36^ The authors suggested up‐regulation in Mas receptors may be due to p38MAPK activity,^36^ but more research is needed to elucidate mechanisms involved in the increase in Mas receptors. To date, there is no research investigating Ang 1‐7 alterations in intracellular Cl^−^, Ca^2+ ^or PKC activity. Based on these findings, Ang 1‐7 likely counteracts Ang‐II‐induced PKC activity and mitochondrial dysfunction based on its ability to preserve CSA and fibre type.

### 2.3 Exercise can attenuate Ang‐II‐induced oxidative damage

Exercise is associated with an increase in health and wellness, which is linked with the decrease in oxidative stress.^37^, ^38^, ^39^, ^40^ Like Ang 1‐7, exercise can be used as a therapeutic outlet for those suffering from illnesses associated with high levels of Ang‐II. Research indicates exercise can combat decreased insulin sensitivity, glucose uptake, fibre type alterations and mitochondrial dysfunction caused by ROS.^37^, ^38^, ^39^ This normalizes skeletal muscle function, producing proper protein degradation and protein synthesis.

Exercise has been shown to alter Ang‐II‐induced effects on Akt to promote proper glucose uptake and insulin sensitivity in skeletal muscle.^37^, ^41^ Exercise could stimulate Akt activity in three ways: through insulin‐like growth factor‐1 (IGF‐1) signal transduction,^37^ increased activity of AMPK^41^, ^42^ or directly reducing oxidative stress.^43^ AMPK might not directly interact with Akt, but increasing AMPK activity may cause an increase in ATP synthesis pathways, which provide ATP for protein synthesis. There has yet to be a link to exercise affecting NOX directly, but studies indicate exercise can increase uncoupling protein 3 (UCP3) activity, which can reduce oxidative stress associated with ROS shifting the protein degradation and synthesis towards normalized values.^12^, ^43^ IGF‐1 signalling is improved by exercise, by increasing IGF‐1/IGF‐1R ratio.^37^ By increasing the IGF‐1/IGF‐1R, muscle can receive protein synthesis signals, which allow skeletal muscle to repair damaged cells and prevent apoptosis. IGF‐1 signal transduction allows improved Akt activity and illustrates exercise inhibitory effect of ROS and Ang‐II‐induced decrease in Akt activity.^9^, ^44^, ^45^ The mechanism for this pathway will be described in Section 3 as this pathway involves more than improving GLUT4 translocation. Whether AMPK directly influences Akt activity is still unclear; however, since Akt is the intersection for many mechanisms, this is certainly worth investigating.

Ang‐II/ROS‐induced loss in muscular function, size and fibre type alterations can be blunted with exercise.^46^ The pathway involving the increase in PGC‐1α is described in Section 2 and is linked to the prevention of muscle loss and dysfunction.^31^, ^42^, ^46^, ^47^, ^48^, ^49^ Research indicates that exercise can prevent ROS‐related muscle damage and reestablish normal phenotype.^38^ Although the experiments have failed to look at ion channel operations in a ROS + exercise model, there may be a link to exercise improving the in/outflow of these channels, as exercise has been shown to improve membrane conductance.^9^

Exercise could be exerting some of these antioxidant effects through Ang 1‐7. Gomes‐Santos 10 indicated exercise could increase Ang 1‐7/Ang‐II ratio and decrease AT_1_R among chronic heart failure rats. This line of research suggests exercise‐related mechanisms can be due in part to the direct manipulation of ACE2/Ang 1‐7/Mas axis. This further suggests the effectiveness of exercise as a therapeutic prescription.

## 3 SECTION 2: UBIQUITIN PROTEASOME SYSTEM

### 3.1 Angiotensin II increases protein degradation and mitochondrial dysfunction

The mechanism of Ang‐II‐induced atrophy via up‐regulation of the ubiquitin proteasome system (UPS) is a complicated and a controversial pathway. Most research indicates that Ang‐II directly stimulates the atrophic state via AT_1_R 13, 18, 47; however, others claim that Ang‐II‐induced atrophy is caused by the up‐regulation of glucocorticoids.^49^ Although the Ang‐II‐induced glucocorticoid pathway leading to atrophy has been refuted 35 both theories have identified AT_1_R activation is required to induce the protein degradation pathways.^49^

Although upstream signalling requires discernment, several studies have confirmed the role of the UPS in Ang‐II‐induced skeletal muscle atrophy. Song 50 used a mouse model infused with Ang‐II for 7 days, found increases in E3‐ligases, p38MAPK activity and decreased Akt phosphorylation in skeletal muscle,^50^ which is similar to Ang‐II action via AT_1_R. Muscle specific E3‐ligases assessed by Song 50 were atrogin‐1 and muscle RING‐finger protein‐1 (MuRF‐1), which are indicative of protein degradation. The increase in p38MAPK activity indicates mitochondrial dysfunction related to an increase in apoptosis, ROS and NF‐κΒ, which stimulates tumour necrosis factor‐alpha (TNF‐α) production.^18^, ^35^, ^51^, ^52^ These proteins cause a down‐regulation of protein synthesis and up‐regulation of protein degradation in skeletal muscle. Phosphorylation of Akt (pAkt) causes an increase in Akt activity, which increases the protein synthesis. However, dephosphorylating pAkt silences protein synthesis, as seen in Ang‐II‐induced pathologies.

**Figure 2:**
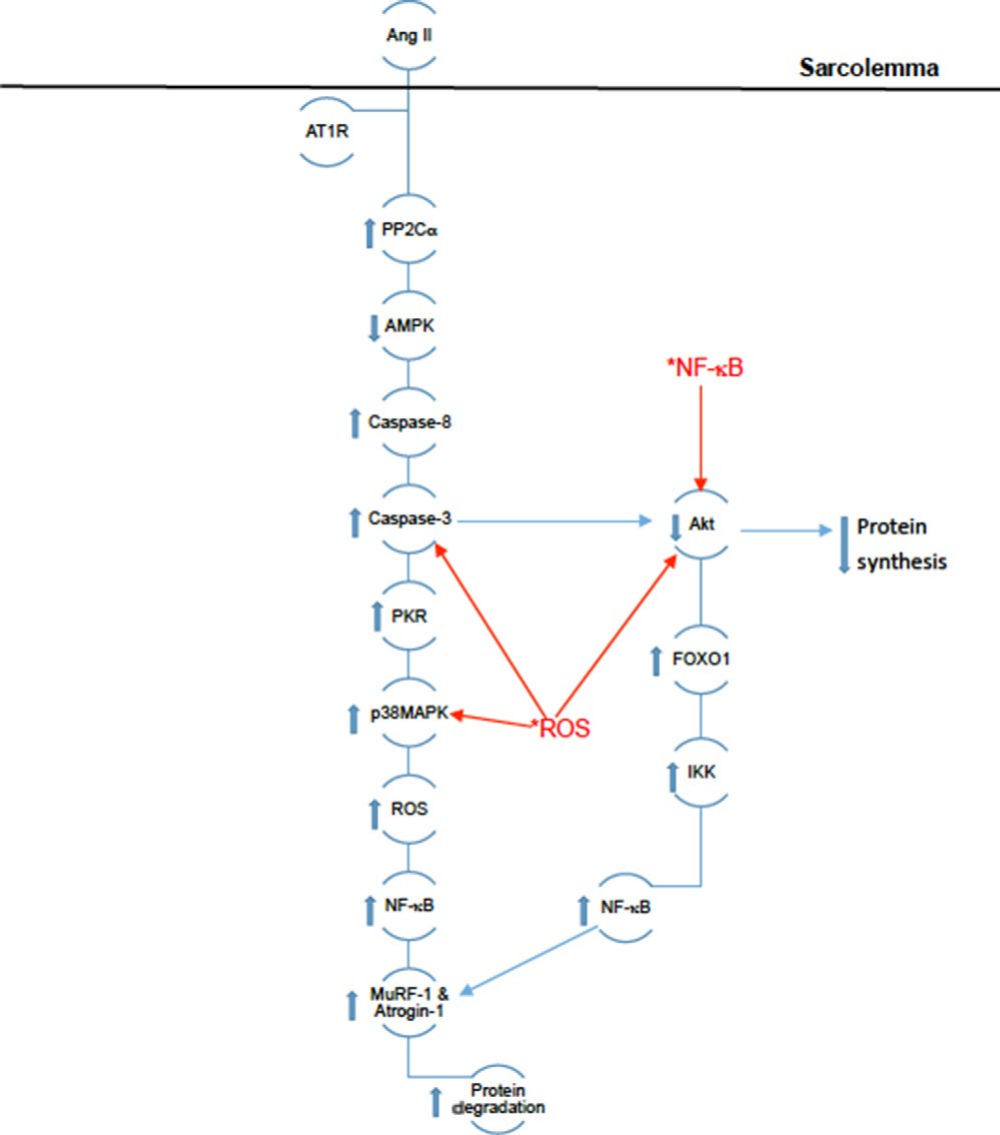
Depicts the theoretical pathway of Ang‐II‐induced increase in protein degradation. *ROS and *NF‐κB indicate their intracellular pre‐existing forms caused by the oxidative stress pathway depicted in Figure 1. *ROS and *NF‐κB and the arrows originating from them indicate proteins they can interact with and illustrate the feedback loops mentioned above. Other arrows illustrate the up‐regulation or down‐regulation of intracellular proteins by Ang‐II mediated by AT1R

The AT_1_R theory suggests that Ang‐II binds to AT_1_R to initiate a cellular response, which involves multiple positive feedback loops that result in skeletal muscle atrophy. AT_1_R activation leads to increased PP2Cα activity, which leads to increased liver kinase B1 (LKB1) activation causing a decrease in AMPK.^31^, ^50^ AMPK represents the intersection of multiple mechanisms, all of which can lead to atrophy. In normal skeletal muscle dephosphorylating AMPK causes a decrease in ATP production,^31^ PGC‐1α and increased Acetyl‐CoA Carboxylase (ACC) 48 and caspase 8 activity.^51^ Decreasing ATP indirectly causes atrophy. If the ATP required for muscular function is not available, then the muscle cell will start degrading MHC in an attempt to produce ATP.^48^ Prolonged ATP scavenging will ultimately lead to apoptosis of skeletal muscle cells. Increased caspase 8 leads to an increase in caspase 3,^51^ which is the next intersection for another atrophic mechanism. Increased caspase 3 leads to decreased Akt activity 4, 5, 50 and increased phosphorylation of protein kinase R (PKR).^51^ Decreased Akt activity allows the increase in forkhead box protein O1 (FOXO1),^40^, ^48^, ^50^, ^53^ which in turn increases I kappa B kinase (IKK) activity producing an influx of NF‐κB, which increases E3‐ligases and protein degradation markers.^35^, ^41^, ^54^ Phosphorylation of PKR also leads to an increase in p38MAPK, which causes an increase in ROS that leads to an increase in NF‐κB.^18^, ^51^ The p38MAPK/ROS complex represents another feedback loop, thus ROS continues to increase p38MAPK activity to produce more ROS. Figure 2 illustrates how Ang‐II can cause an imbalance between protein degradation and synthesis resulting in atrophy and indicates the steps intracellular ROS can affect. Taking into consideration that Ang‐II can stimulate ROS through NOX, these atrophic mechanisms can be initiated through various pathways and possibly have the ability to skip steps. So inhibiting the decrease in AMPK or the increased NOX activity will not completely inhibit atrophy.

The complexity of the mechanisms leading to atrophy represents the difficulty in proper treatment, which is why many treatments involve ACE or AT_1_R inhibition in an attempt to prevent these mechanisms leading to atrophy.

### 3.2 Angiotensin 1‐7 inhibits p38MAPK and IKK and decreases protein degradation activity

Little is known regarding mechanisms underlying the downregulation of protein degradation via Ang 1‐7/MasR in skeletal muscle. Application of Ang 1‐7 in a unilateral immobilized atrophic mouse model has shown to inhibit Ang‐II‐induced increases in E3‐ligases MuRF‐1 and atrogin‐1.^55^ Ang 1‐7 prevents p38MAPK phosphorylation and increases Akt activity, which are both downstream from caspase 3.^11^, ^33^ However, when considering the feedback loops in relation to the protein degradation, the activity or inactivity from caspase 3, p38MAPK and Akt, Ang 1‐7 must interact with proteins other than NOX.

Ang 1‐7, like Ang‐II, may have a wide range of effects on the cell, which is indicative of the anti‐atrophic effects. Mechanisms involved in Ang 1‐7 anti‐atrophic abilities have not been established in the skeletal muscle. Ang 1‐7 can inhibit increases in IKK activity, E3 ligases 55, 56 and prevent p38MAPK phosphorylation as well as increase Akt activity in skeletal muscle.^11^, ^33^, ^57^ As discussed previously, ROS alone can induce protein degradation through these proteins, which might suggest that Ang 1‐7 inhibitory effects are related to ROS suppression. To truly understand the mechanism of Ang 1‐7, future research should analyse the degree of protein degradation through the inhibition of ROS production via Ang 1‐7 and ML171 (selective NOX1 inhibitor). If total protein degradation is inhibited by ML171 and Ang 1‐7 separately, this would mean that protein degradation is ROS dependent and the Ang 1‐7 mechanism ends with the inhibition of NOX.

### 3.3 Exercise promotes ATP generating pathways

Exercise can inhibit protein degradation in a similar fashion as Ang 1‐7. As previously noted, exercise can decrease intracellular ROS, which can activate a wide variety of pathogenic mechanisms, such as protein degradation and fibrosis. Along with the decrease in intracellular ROS, exercise can promote AMPK activity in skeletal muscle.^42^, ^58^ Increased activity of AMPK can result in the promotion of ATP generating pathways by increasing fatty acid oxidation and glucose uptake. This process counteracts the Ang‐II pathogenic phenotype, which involves a decrease in available ATP within skeletal muscle. Increases in oxidative metabolism are likely a result of AMPK's ability to increase PGC‐1α activity.^42^ Palacios 42 indicated that exercise could lead to an increase in sirtuin 3 (SIRT3), which can up‐regulate PGC‐1α by phosphorylating cAMP response element‐binding protein (CREB). PGC‐1α can alter skeletal muscle fibre’s metabolic properties shifting towards more aerobic‐based metabolism. This shift also counteracts Ang‐II's effects, where Ang‐II decreases PGC‐1α causing both muscle fibres types to rely primarily on anaerobic metabolic pathways for ATP production. This process can lead to a decrease in exercise capacity,^15^ which allows this pathogenesis to continue uninhibited. The effects of exercise on AMPK are likely not a result of up‐regulation of the ACE2/Ang 1‐7/Mas axis, as Ang 1‐7 has yet to be linked to the up‐regulation of AMPK.

Koltai 46 demonstrated that sirtuin 1 (SIRT1) activity is increased by exercise through the increase of nicotinamide adenine dinucleotide/nicotinamide phosphoribosyltransferase (NAD^+^/NAMPT). Although PGC‐1α levels were not accounted for in this study, it is a possible target for SIRT1 as well as NF‐κB and myogenic differentiation antigen (MyoD), which are linked to proteolysis and muscle wasting.^46^ Koltai's 46 investigation indicated that exercise leads to an increase in UCP3 and decrease hypoxia‐inducible factor 1‐alpha (HIF‐1α) and VEGF. HIF‐1α and VEGF overexpression are linked to tumour growth and is up‐regulated by ROS.^46^ Ang‐II‐related pathologies have not been associated with HIF‐1α or VEGF yet, but this study provides a possible link to the Ang‐II pathogenesis.

## 4 SECTION 3: PROTEIN SYNTHESIS

### 4.1 Angiotensin II inhibits Akt and may interact with p53

Along with an increase in protein degradation, Ang‐II‐induced pathogenesis impacts protein synthesis. Ang‐II inhibits Akt, which increases E3‐ligases causing an imbalance between protein synthesis and degradation. The decrease in Akt activity may be caused by the decrease in IGF‐1R associated with ROS and NF‐κB, which prevents IGF‐1 signal transduction from phosphorylating Akt preventing protein synthesis to occur. Furthermore the increase in ROS can also directly reduce IGF‐1R expression.^20^, ^21^, ^23^

As discussed earlier AMPK activity is inhibited by Ang‐II, but under normal conditions, the increase in AMPK activity can inhibit protein synthesis to promote ATP production. Inhibition of AMPK increases caspase‐3 activity, which may inhibit Akt. However, there is an overlap between Ang‐II and p53 pathways because p53 also decreases protein synthesis and increases degradation pathways.^59^ p53 can bind to AT_1_R and cause an increase in Ang‐II establishing a link between mechanisms.^59^ It would seem p53 could play a pivotal role in the pathogenic cascade of Ang‐II but the interaction between Ang‐II and p53 is not yet established.

### 4.2 Angiotensin 1‐7 improves IGF‐1/IGF‐1R signal transduction

**Figure 3:**
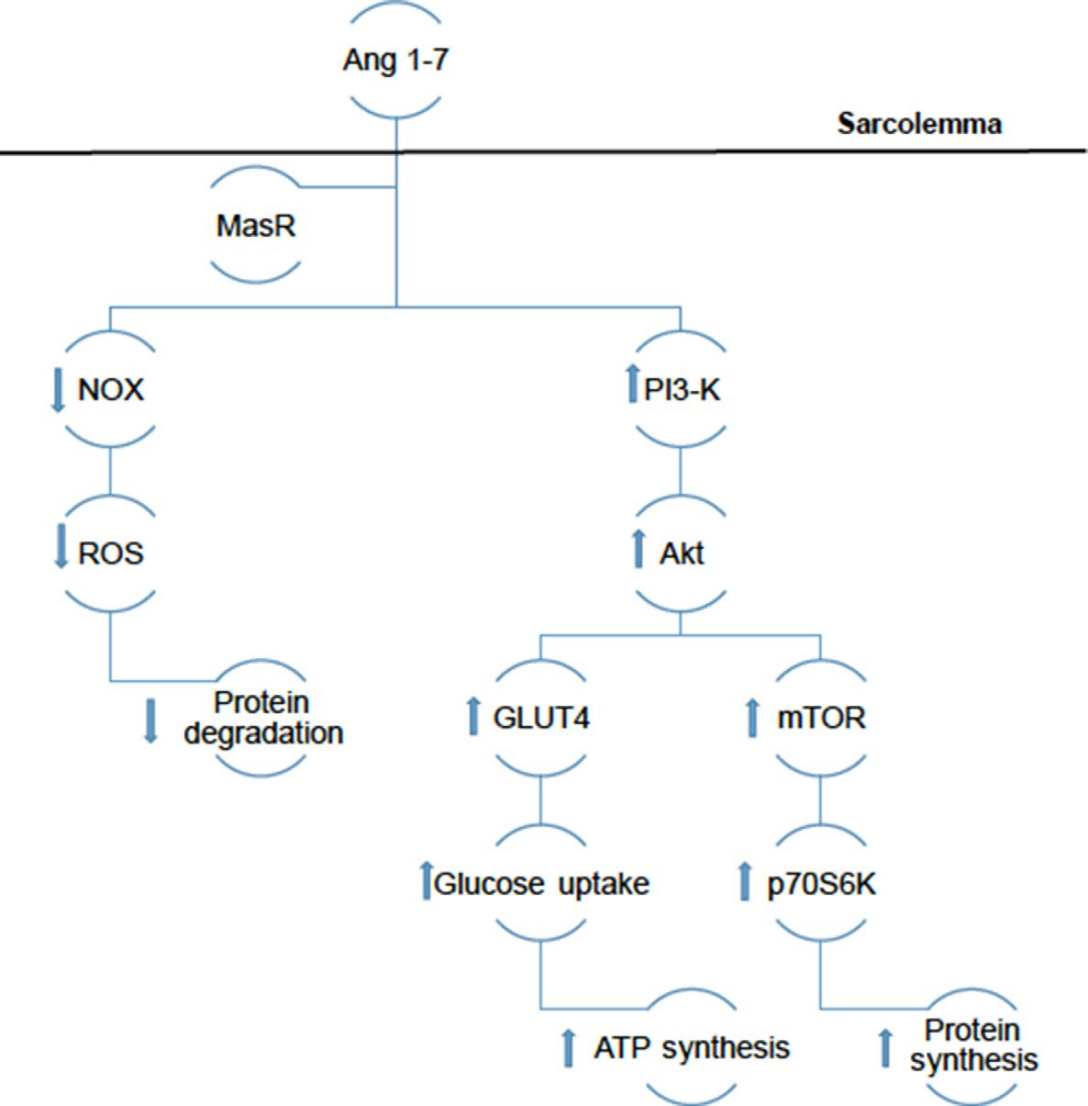
Illustrates the theorized mechanisms of Ang 1‐7 in regards to the down‐regulation of ROS production and up‐regulation of protein synthesis. The arrows indicate the up‐regulation or down‐regulation of intracellular proteins by Ang 1‐7 mediated by MasR

As discussed previously, Akt activity plays an important role in protein synthesis. Ang 1‐7 improves IGF‐1 signal transduction by increasing IGF‐1R through the increase in Akt activity, which increases protein synthesis. Increased IGF‐1 signalling increases PI3K activity, which in turn increases Akt activity. The activation of Akt inhibits FOXO1 activity causing a decrease in protein degradation and increases in mammalian target of rapamycin (mTOR) activity,^60^ which leads to increased protein synthesis via increased levels of P70S6K. Inhibiting FOXO1 prevents the increase in IKK activity and translocation of NF‐κB. Akt activity also leads to an inhibition of glycogen synthase kinase‐3 beta (GSK‐3β), which down‐regulates glycogen synthase activity and promotes protein synthesis.^22^ Section 1 noted the ability for Akt to allow GLUT4 translocation leading to improved glucose sensitivity and influx of glucose into skeletal muscle. This influx of glucose produces ATP via glycolysis. ATP production provides the energy that is needed for protein synthesis to occur. Under Ang‐II‐induced pathology, ATP is depleted which drives the offset between protein degradation and synthesis. Although actions of Ang 1‐7 are still unclear, the signal transduction of Ang 1‐7 might be able to inhibit AMPK and further promote protein synthesis. Figure 3 illustrates Ang 1‐7 mechanisms inhibiting NOX activity, increasing ATP production and protein synthesis.

### 4.3 Exercise improves autophagy regulation

Like Ang 1‐7, Exercise improves Akt activity via IGF‐1 signal transduction, which improves protein synthesis.^22^ Exercise improves autophagy regulation through Akt/FOXO3 signalling.^37^ This allows skeletal muscle cells to properly dispose of damaged muscle tissue caused by exercise. Whereas, Ang‐II induces an up‐regulation of apoptosis and a dysfunctional autophagy process.^37^, ^58^ Exercise further promotes proper blood flow, which attenuates Ang‐II‐induced vasoconstriction. Ang‐II‐induced vasoconstriction can promote an increase in oxidative stress leading to tissue destruction.^61^ Alleviating this restricted blood flow allows the cellular activity to function properly. Cunha 38 demonstrated that exercise achieves a normalization between protein degradation and synthesis, which was associated with the decline in oxidative stress. Exercise also increased p53 mRNA expression,^58^ further suggesting exercise's role in maintaining a balance between protein synthesis and degradation and a link between p53 and the RAS axes.

## 5 SECTION 4: FIBROSIS

### 5.1 Angiotensin II‐induced mitochondrial dysfunction increases ERK1/2 activity

The loss of muscle coincides with fibrotic deposition; Ang‐II induces atrophy while simultaneously inducing fibrosis. As muscle is degraded, fibrotic factors are deposited, making the skeletal muscle more tendinous, which results in the loss of function and force production. Fibrosis contributes to the progression of muscle loss and function and is characterized by accumulation of extracellular matrix (ECM). The Ang‐II‐induced fibrosis can produce multiple proteins that continuously stimulate fibrotic proteins.

Ang‐II primarily drives fibrosis through NOX‐derived ROS 32; ROS increases p38MAPK activity which in turn increases transforming growth factor‐beta (TGF‐β) expression.^6^ The increase in p38MAPK coincides with an increase in extracellular signal‐regulated kinase 1/2 (ERK1/2) activity.^6^ ERK1/2 has been linked to a variety of cellular responses but with regards to fibrosis, ERK1/2 is a suggested link to an increase in fibronectin, collagen and TGF‐β synthesis.^6^, ^7^ ERK1/2 also activates the Smad2 and Smad3 complex, which forms a complex with Smad4 and translocates to the nucleus.^45^ The translocation of Smad4 to the nucleus causes an increase in micro RNA‐21 (miR‐21), plasminogen activator inhibitor‐1 (PAI‐1) and single mothers against decapentaplegic 7 (Smad‐7).^7^, ^45^, ^62^ The miR‐21 further stimulates the production of TGF‐β. PAI‐1 has been suggested to progress Ang‐II‐related diseases by stimulating TGF‐β and TNF‐α.^63^ PAI‐1 has been implicated in the reduction of plasmin, which has been associated with the up‐regulation of matrix metalloproteinases (MMP). Thus the inhibition of MMPs allows unregulated fibrotic deposition. Furthermore, Smad7 is associated with inhibiting both TGF‐β signal transduction and NF‐κB translocation. However, the role of Smad7 in fibrotic pathogenesis is unclear, but the inhibition of TGF‐β signal transduction will not prevent fibrosis in skeletal muscle in a pathological state. TGF‐β stimulates myofibroblasts to produce fibronectin, collagen and connective tissue growth factor (CTGF). Furthermore, this protein has been found to reduce Mas receptors in myofibroblasts but not in myoblasts,^8^ suggesting that TGF‐β can act on different cell types to induce fibrosis and indicates the importance of the non‐classical RAS axis in the inhibition of fibrosis. The versatility of TGF‐β allows it to compound the actions of Ang‐II.

TGF‐β can stimulate skeletal muscle cells along with Ang‐II to further enhance fibrosis. TGF‐β has been found to increase ROS production and provides a strong stimulus for the generation CTGF. CTGF has also been shown to induce fibrosis through the interaction with AT_1_R. In addition to the ability of TGF‐β to contribute to the production of fibrotic factors, TGF‐β can also stimulate NOX‐activated ROS and contribute to atrophy.^6^, ^32^, ^62^, ^64^, ^65^

### 5.2 Angiotensin 1‐7 reduces fibrotic factors

Fibrotic deposition is regulated by the actions of Ang 1‐7 and contributes to anti‐fibrotic process through the inhibition of NOX. By preventing ROS production, Ang 1‐7 decreases activated ERK1/2 proteins thus preventing synthesis of fibrotic agents. Studies have indicated that Ang 1‐7 can reduce fibronectin, CTGF, miR‐21, TGF‐β, collagen I/III and Smad complex.^7^, ^66^, ^67^ This therapeutic pathway is mediated by Mas receptors. One study using Mas‐knockout mice indicated more enhanced fibrosis compared to fibrosis‐induced in mice expressing Mas receptors.^45^ Mentioned earlier was the ability of TGF‐β to reduce Mas expression in myofibroblasts; application of Ang 1‐7 prevented this reduction by increasing Akt activity.^8^ The mechanism most likely involves the inhibition of TGF‐β synthesis; however, the authors did not cite Akt activity inhibiting TGF‐β synthesis.

Dystrophic models have been shown to have higher levels of ACE2.^68^ ACE2 converts Ang‐II into Ang 1‐7, indicating that the body's natural response to this fibrosis‐inducing disease is to alter the state of Ang‐II. ACE2 supplementation helped to reduce fibrotic factors and Ang 1‐7 infusion caused an increase in ACE2.^68^ Cellular activity caused by Ang 1‐7 goes beyond the inhibition of ROS production to prevent Ang‐II‐induced diseases. Clearly, the non‐classical RAS axis is an important pathway to understand and establish a remission of Ang‐II‐induced diseases.

### 5.3 Exercise increases SOD and myofibres which can prevent fibrotic deposition

Fibrosis can be regulated by exercise by improving oxidative properties of skeletal muscle and by inhibiting the actions of NF‐κB, which stimulates an increase in the inflammatory cytokine TNF‐α.^41^, ^69^, ^70^, ^71^ Inflammation is an indication of muscular remodelling, which exercise can regulate by increasing in I‐kappa‐B‐beta/alpha (IKB‐β/α), which actively inhibits NF‐κB activity.^41^ This indicates exercise as a logical way to restrict the pathogenic actions of Ang‐II.

As stated previously, exercise also regulates the expression of AT_1_R, which can inhibit an overstimulation caused by Ang‐II and CTGF under fibrotic conditions. This allows for regulation of fibrotic protein synthesis as well as ROS production, further providing a normalization of skeletal muscle remodelling. Furthermore, lifelong exercise has been linked to higher resting levels of superoxide dismutase (SOD).^69^ SOD acts as a superoxide scavenger and reduces damage caused by ROS, which is a key component to the pathological process of Ang‐II in skeletal muscle.

Exercise is usually looked at as metabolic training or strength training; however, stretching can be used as a form of exercise as well. Hwang 72 used a muscular laceration rat model to identify the effects of passive stretching on fibrosis prevention. This study indicated passive stretching as a plausible therapy to decrease fibrosis. Treatment groups had increased the number of new myofibres, fibre diameter and strength.^72^ Although the model used did not have Ang‐II‐induced fibrosis, this study does provide an alternative way to exercise to produce anti‐Ang‐II‐induced pathologies. Future research should investigate if/how stretching can reduce Ang‐II‐induced pathological effects because this would be a good tool to use for those who might be immobile or who have muscular dystrophy.

## 6 CONCLUSION

Ang‐II disrupts skeletal muscle function by increasing NOX‐derived ROS leading to the imbalance between protein degradation and synthesis. The muscle lost due to atrophy is replaced by fibrotic deposition, which further impairs skeletal muscle function. Ang 1‐7 and exercise work to normalize and maintain skeletal muscle function. Ang 1‐7 has been shown to inhibit Ang‐II stimulation of NOX, which prevents ROS production allowing for normal skeletal muscle functioning. Exercise increases antioxidant enzymes, which decreases intracellular ROS. Exercise also increases PGC‐1α activity, which improves skeletal muscle oxidative metabolism. Improved oxidative metabolism allows the muscle to clear ROS and ultimately reduce oxidative stress‐related damage.

Future research should investigate how the RAS axes are affected in skeletal muscle by long‐term exercise and Ang 1‐7. Overall, more research is needed to properly identify the mechanism related to the therapeutic role of Ang 1‐7 and exercise within skeletal muscle. Currently no research has investigated Ang 1‐7 or exercise effects on ion channels within skeletal muscle under Ang‐II‐induced pathology. This could provide better treatment strategies for patients suffering from Ang‐II‐induced pathologies. Current treatments typically consist of angiotensin receptor blockers (ARBs) or ACE inhibitors, which are good for short‐term prevention but do not make lasting physiological changes to the skeletal muscle such as exercise would.

Based on the how AMPK is regulated among normal‐functioning skeletal muscle, Ang‐II must inhibit crosstalk pathways. When normal skeletal muscle cells have low ATP levels, AMPK activity increases, which inhibits pathways that require ATP‐like protein synthesis. However, as stated in this review, Ang‐II inhibits both AMPK and the protein synthesis pathways regardless of the availability of ATP. Also, Ang‐II has been linked to the increase in LKB1, which under normal conditions increases AMPK activity. The interactions between Ang‐II and AMPK might involve p53 activity but this pathway remains unclear and more research is needed to identify how Ang‐II is able to produce conflicting signals. Understanding the mechanisms involved in this pathway is important for future pharmaceutical production.

This review identified the mechanisms of Ang‐II, Ang 1‐7 and exercise and how they affect skeletal muscle atrophy. Ang 1‐7 antagonizes the actions of Ang‐II and in some reports increase hypertrophy producing improved skeletal muscle function. Exercise can alter the physiological state of skeletal muscle and increase Ang 1‐7, which allows the muscle to decrease the actions of Ang‐II‐induced pathologies.

## CONFLICTS OF INTEREST

The authors confirm that there are no conflicts of interest.

## AUTHOR CONTRIBUTION

Mark Winslow wrote the paper, Stephanie Hall PhD revised paper.
